# Characterization of the male *Apc^Min/+^* mouse as a hypogonadism model related to cancer cachexia

**DOI:** 10.1242/bio.20136544

**Published:** 2013-11-04

**Authors:** James P. White, Melissa J. Puppa, Aditi Narsale, James A. Carson

**Affiliations:** Integrative Muscle Biology Laboratory, Department of Exercise Science, Public Health Research Center, University of South Carolina, Columbia, SC 29208, USA

**Keywords:** Testosterone, Hypogonadism, Muscle, Cancer cachexia

## Abstract

Cancer cachexia, the unintentional loss of lean body mass, is associated with decreased quality of life and poor patient survival. Hypogonadism, involving a reduction in circulating testosterone, is associated with the cachectic condition. At this time there is a very limited understanding of the role of hypogonadism in cancer cachexia progression. This gap in our knowledge is related to a lack of functional hypogonadal models associated with cancer cachexia. The *Apc^Min/+^* mouse is an established colorectal cancer model that develops an IL-6 dependent cachexia which is physiologically related to human disease due to the gradual progression of tumor development and cachexia. The purpose of this study was to assess the utility of the *Apc^Min/+^* mouse for the examination of hypogonadism during cancer cachexia and to investigate if IL-6 has a role in this process. We report that *Apc^Min/+^* mice that are weight stable have comparable testosterone levels and gonad size compared to wild type mice. Cachectic *Apc^Min/+^* mice exhibit a reduction in circulating testosterone and gonad size, which has a significant association with the degree of muscle mass and functional strength loss. Circulating testosterone levels were also significantly associated with the suppression of myofibrillar protein synthesis. Skeletal muscle and testes androgen receptor expression were decreased with severe cachexia. Although testes STAT3 phosphorylation increased with severe cachexia, systemic IL-6 over-expression for 2 weeks was not sufficient to reduce either testes weight or circulating testosterone. Inhibition of systemic IL-6 signaling by an IL-6 receptor antibody to *Apc^Min/+^* mice that had already initiated weight loss was sufficient to attenuate a reduction in testes size and circulating testosterone. In summary, the *Apc^Min/+^* mouse becomes hypogonadal with the progression of cachexia severity and elevated circulating IL-6 levels may have a role in the development of hypogonadism during cancer cachexia.

## Introduction

Cancer induced cachexia is a condition of unintentional body weight loss associated with the loss of skeletal muscle with or without the loss in adipose tissue (Tisdale, 2010). The complex nature of cachexia is associated with systemic disorders that include chronic inflammation, insulin resistance, anemia, anorexia and hypogonadism (Evans et al., 2008). While some of these systemic disorders associated with wasting, such as chronic inflammation and insulin resistance, have been and are being widely investigated (Argilés et al., 2009; Carson and Baltgalvis, 2010; Fearon et al., 2012), there remain gaps in our understanding of hypogonadism's regulatory role in the progression of cancer cachexia. Hypogonadism has potential to be a contributor to cachexia progression directly and also through interactions with inflammation, anemia, and functional impairments (Burney et al., 2012; Del Fabbro et al., 2010). A better mechanistic understanding of hypogonadism's function in the progression of cancer cachexia will define regulatory pathways that can be manipulated therapeutically for improved treatment options. While castration and androgen replacement mouse models are valuable for understanding testosterone's anabolic action, they fall short in replicating the disrupted systemic environment created by cancer. A barrier in our understanding of testosterone action during cancer cachexia has been directly associated with tumor implant models that induce create a rapid and severe cachexia, but do not allow for the physiological examination of the hypogonadal state (Murphy et al., 2012).

Testosterone is a potent regulator of muscle mass, and circulating levels decrease in the hypogonadal state. The anabolic effects of testosterone supplementation are observed in young and old men in a dose dependant manner (Bhasin et al., 2001; Bhasin et al., 2005). In contrast, low testosterone is associated with muscle wasting and reduction in functional strength with (Garcia et al., 2006; Grinspoon et al., 1996) or without (Katznelson et al., 1996; Mauras et al., 1998) an underlying disease. Furthermore, several human diseases associated with a loss of lean body mass such as diabetes (Dhindsa et al., 2004), chronic obstructive pulmonary disorder (COPD) (Van Vliet et al., 2005), and HIV-AIDS (Cohan, 2006) have reported a reduction in circulating testosterone in patients. Due to the anabolic properties of testosterone and its pharmacological derivatives, a strong research focus has been given to maintain anabolic hormones during catabolic conditions (Kadi, 2008; Laaksonen et al., 2003; Simon et al., 2001). Furthermore, testosterone and other anabolic steroids have been shown to be effective in rescuing the loss of muscle mass in wasting conditions (Bhasin and Storer, 2009). Although animal models of androgen ablation are useful to study effects on muscle biology (White et al., 2012) the underlying relevance of low testosterone is not well understood during disease states. Currently there are no reports on testosterone levels or gonadal function during cancer cachexia in the mouse. This might be due to the lack of attention given to the role of hypogonadism in cachexia or traditional tumor inoculation models do not develop hypogonadism.

The *Apc^Min/+^* mouse is an established model of colorectal cancer and cachexia (Baltgalvis et al., 2008; White et al., 2011b). An advantage of this mouse model over other models of experimental cachexia is the gradual progression of tumor development and muscle wasting that is more physiologically related to human disease, when compared to tumor implant models. Tumor implant models result in a disproportionate tumor mass in relation to body mass, which can create rapid muscle wasting related to amplified systemic inflammatory and metabolic disruptions. Although implant studies can be carried out for several weeks, careful examination of these studies demonstrates that the weight loss and muscle mass loss often occurs in just several days. It has been clearly demonstrated that fasting a mouse for just 24 hours can create greater than 10% body weight loss (Ayala et al., 2006), and this condition does not replicate the physiologic advancement of cancer cachexia. The *Apc^Min/+^* mouse demonstrates a sustained and persistent weight loss over at least 4–5 weeks (Puppa et al., 2011a; White et al., 2011b) that provides a model for physiologic examination of systemic disruptions, such as hypogonadism. Work from our laboratory has shown the severity of cancer development and cachexia in the *Apc^Min/+^* mouse is dependent on the cytokine IL-6 (Baltgalvis et al., 2008; White et al., 2011b), which is also thought to be a factor in the development of human cachexia. The role of hypogonadism during the progression of cachexia in the *Apc^Min/+^* mouse has not been established. The purpose of this study is to determine the utility of the *Apc^Min/+^* mouse as a model to study hypogonadism during cancer cachexia. Our research question was to determine if a hypogonadal state was associated with the progression of muscle mass loss in the *Apc^Min/+^* mouse. Furthermore, we examined if this condition was associated with circulating IL-6 levels. Our results demonstrate that the *Apc^Min/+^* mouse is a functional model for the study of hypogonadism during cancer cachexia.

## Results

### Circulating testosterone is reduced during the development of cachexia in the *Apc^Min/+^* mouse

**Fig. 1.**
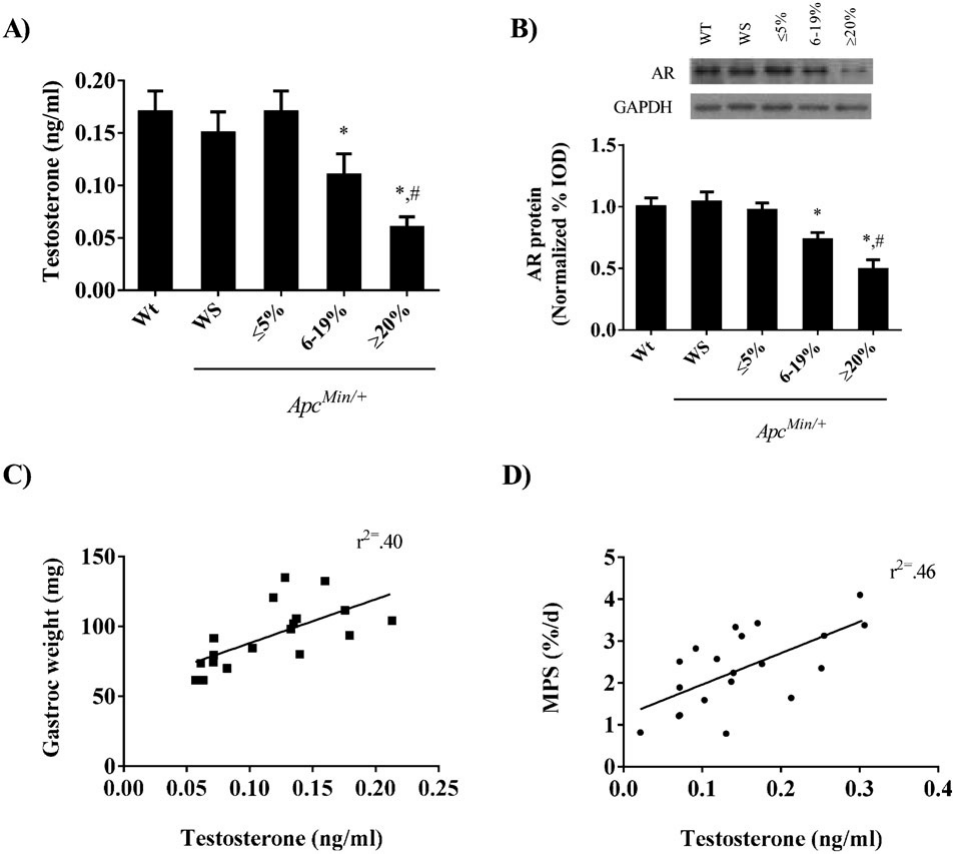
The reduction in circulating testosterone correlates with several parameters of cachexia in the *Apc^Min/+^* mouse. (A) Circulating testosterone throughout the progression of cachexia. (B) *Upper* representative Western blot of the androgen receptor in the gastrocnemius. *Lower* quantified androgen receptor expression throughout the progression of cachexia in the gastrocnemius muscle. (C) Correlation between circulating testosterone and gastrocnemius weight (gastroc). (D) Correlation between circulating testosterone and myofibrillar protein synthesis. Values are means ± SE. *Signifies difference from wild-type mice. # Signifies difference from mice with 6–19% body weight loss.

Circulating testosterone was measured throughout the progression of cachexia. We found no difference in circulating testosterone between wild-type and weight stable *Apc^Min/+^* mice or *Apc^Min/+^* mice initiating body weight loss (Fig. 1A). As the severity of cachexia progressed circulating testosterone decreased. Compared to weight stable *Apc^Min/+^* mice there was a 27% reduction in testosterone during moderate body weight loss and a 60% reduction in mice with severe weight loss. Androgen receptor expression, a maker of cellular androgen bio activity was similar between wild-type mice and weight stable *Apc^Min/+^* mice and *Apc^Min/+^* mice initiating body weight loss (Fig. 1B). Similar to what was observed with circulating testosterone, muscle androgen receptor expression was reduced 25% and 50% in *Apc^Min/+^* mice with moderate and severe body weight loss respectively. There were positive correlations between circulating testosterone and gastrocnemius muscle mass (Fig. 1C, r^2^ = 0.40, *P*<0.05) and myofibrillar protein synthesis (Fig. 1D, r^2^ = 0.46, *P*<0.05).

### Testicular atrophy corresponds with the reduction in circulating testosterone

**Fig. 2.**
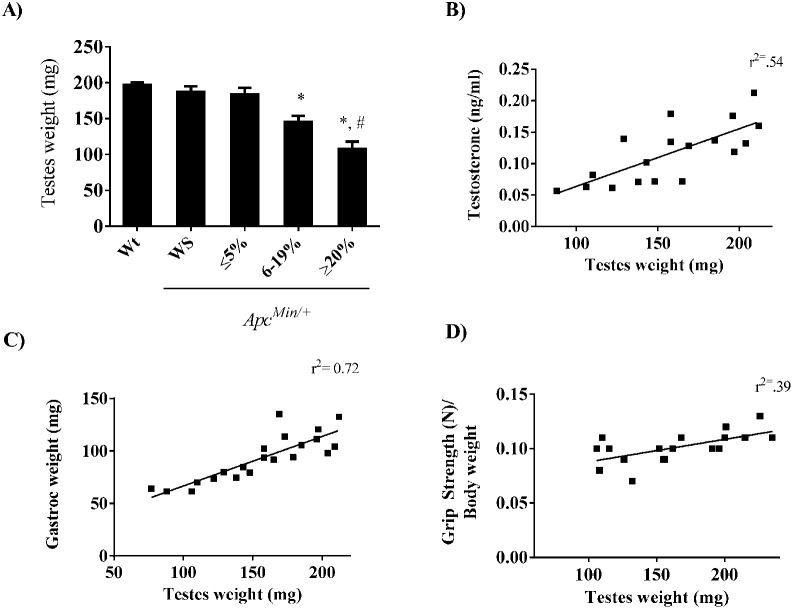
Gonadal atrophy during cachexia. (A) Testes weight throughout the progression of cachexia. Correlation between testes weight and (B) testosterone, (C) gastrocnemius muscle (gastroc) weight and (D) grip strength. Values are means ± SE. *Signifies difference from wild-type mice. # Signifies difference from mice with 6–19% body weight loss.

**Fig. 3.**
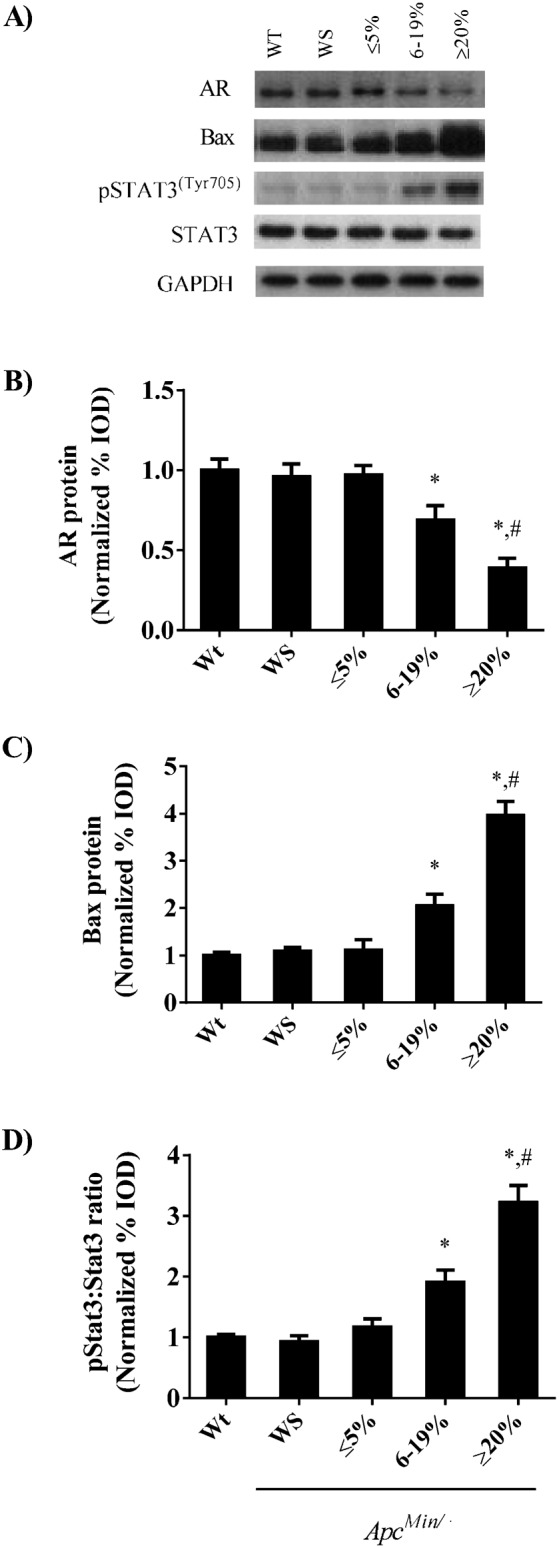
(A) Representative Western blot of the androgen receptor, Bax, phosphorylated and total STAT3 in the testes. Quantified (B) androgen receptor, (C) Bax and (D) Ratio of phospho and total STAT3 expression throughout the progression of cachexia. Values are means ± SE. *Signifies difference from wild-type mice. # Signifies difference from mice with 6–19% body weight loss.

Testicular atrophy was observed in the *Apc^Min/+^* mice during the progression of cachexia. Testes mass was similar in wild-type and weight stable *Apc^Min/+^* mice and mice with initial body weight loss (Fig. 2A). *Apc^Min/+^* mice with moderate and severe cachexia had 22% and 42% reduction in testes mass respectively. The reduction in testes mass correlated with the decrease in circulating testosterone (Fig. 2B, r^2^ = 0.54, *P*<0.05) and gastrocnemius muscle mass (Fig. 2C, r^2^ = 0.72, *P*<0.05). In addition, there was a positive correlation between grip strength and testes mass in *Apc^Min/+^* mice (Fig. 2D, r^2^ = 0.39, *P*<0.05). In conjunction with testicular atrophy, we observed a reduction in testes androgen receptor expression during the progression of cachexia (Fig. 3A). While no differences were observed between wild-type and weight stable *Apc^Min/+^* mice or *Apc^Min/+^* mice initiating body weight loss, there was a 30% reduction in androgen receptor expression in *Apc^Min/+^* mice exhibiting moderate body weight loss and 60% reduction in mice with severe weight loss. Expression of the pro-apoptotic Bax protein corresponded to the induction of testes atrophy as expression was increased 2 fold in *Apc^Min/+^* mice with moderate weight loss and roughly 4 fold in *Apc^Min/+^* mice with severe weight loss (Fig. 3B). Testes STAT-3 activation, a marker of IL-6 signaling was increased by 91% and roughly 3 fold in *Apc^Min/+^* mice with moderate and severe body weight loss respectively (Fig. 3C). There were no differences in STAT-3 activation between non cachectic groups.

### Circulating IL-6 regulates the development of hypogonadism in the *Apc^Min/+^* mouse

**Fig. 4.**
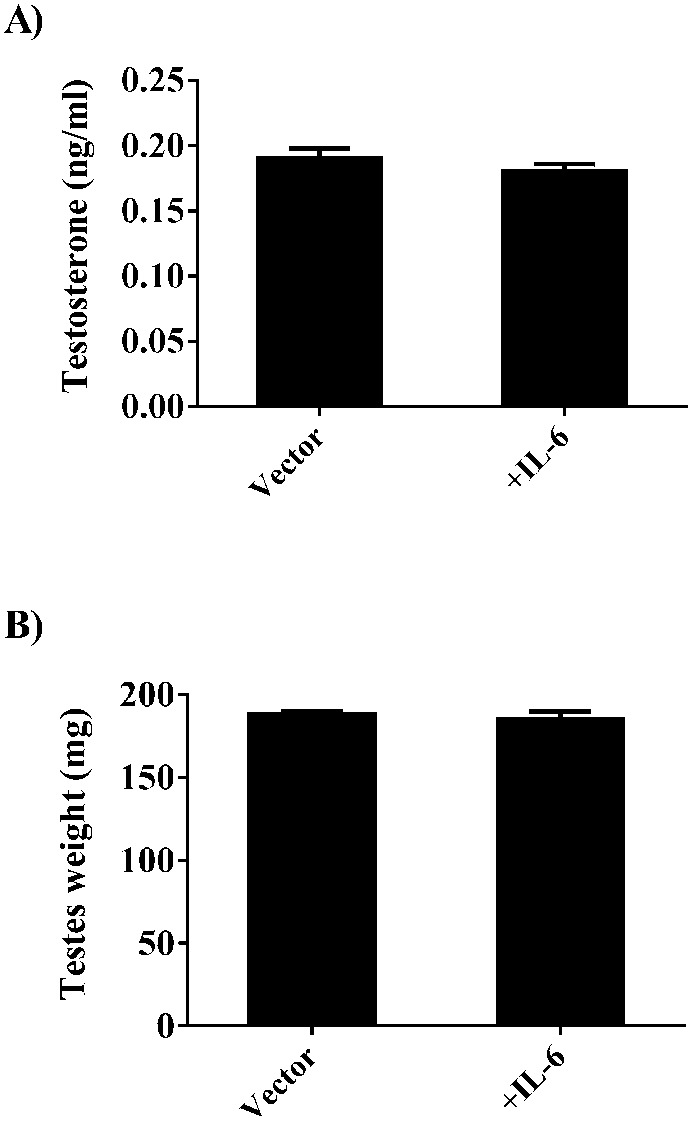
Acute IL-6 over-expression does not induce hypogonadism. (A) Circulating testosterone and (B) testes weight in mice over-expressing systemic IL-6 or control vector. Values are means ± SE.

**Fig. 5.**
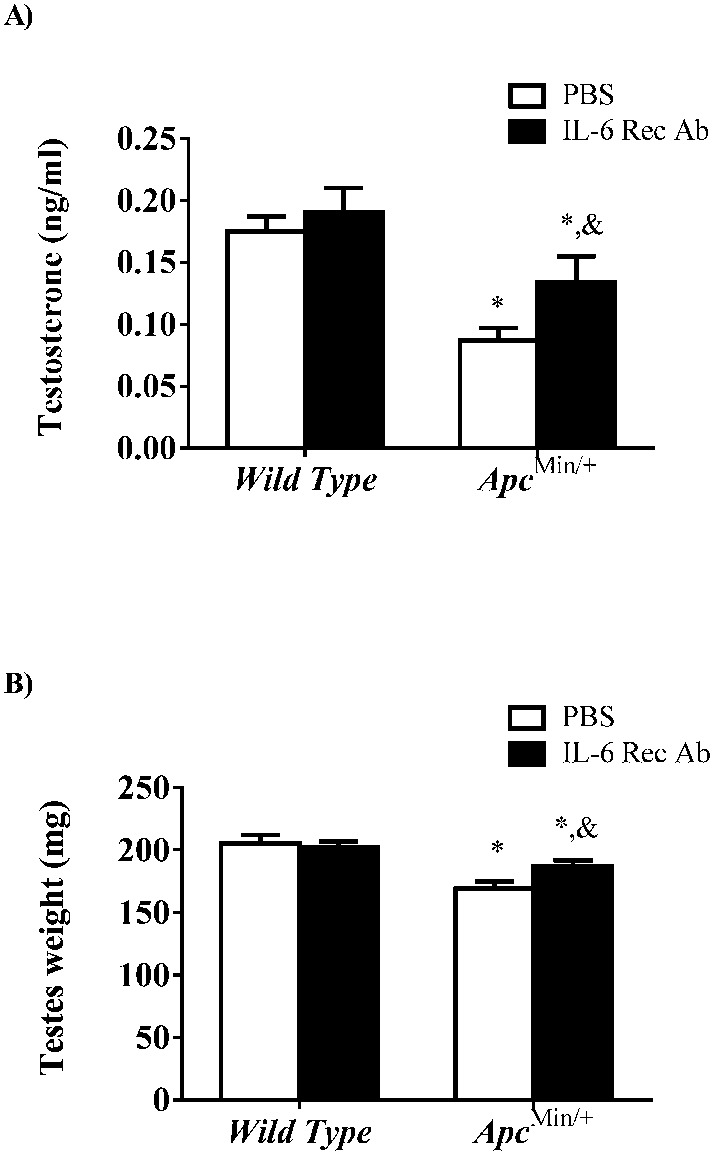
Inhibition of IL-6 signaling attenuates the development of hypogonadism in the *Apc^Min/+^* mouse. (A) Circulating testosterone and (B) testes weight in mice treated with an IL-6 receptor antibody (IL-6Rec Ab) or PBS control. Values are means ± SE. *Signifies difference from PBS treated wild-type mice. & Signifies difference from PBS treated *Apc^Min/+^* mice.

To explore the direct effects of IL-6 on the development hypogonadism in the *Apc^Min/+^* mouse we over-expressed circulating IL-6 for two weeks in weight stable *Apc^Min/+^* mice. The magnitude of IL-6 over-expression in this study was similar to what is observed in severely cachectic *Apc^Min/+^* mice (Puppa et al., 2011b; White et al., 2011b). IL-6 over-expression had no effect on circulating testosterone (Fig. 4A) or testes mass (Fig. 4B) in the *Apc^Min/+^* mouse. We next treated *Apc^Min/+^* mice exhibiting initial body weight loss with an IL-6 receptor antibody for two weeks to block systemic IL-6 signaling including both classical and trans IL-6 signaling (Rose-John, 2012), during a pivotal time point in the progression of cachexia. Treatment with the IL-6 receptor antibody attenuated the decline in circulating testosterone (Fig. 5A) and testicular atrophy (Fig. 5B). Compared to wild-type mice, the PBS treated *Apc^Min/+^* mouse decreased circulating testosterone roughly 50%, while treatment with the IL-6 receptor antibody limited the decrease to a 23% reduction in testosterone (Fig. 5A). Testes mass decreased 17% in the PBS treated mice while IL-6 antibody treatment reduced this to 8% loss in testes mass (Fig. 5B). Both testosterone levels and testes mass did not return to wild-type values. There was no effect of the IL-6 receptor antibody on circulating testosterone or testes mass in the wild-type mice.

**Fig. 6.**
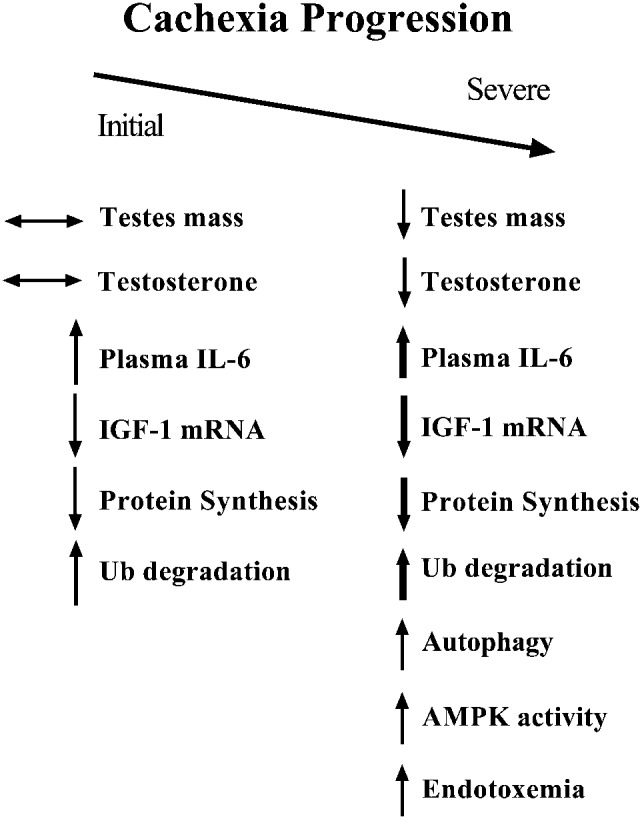
Summary of inflammatory, systemic and tissue specific changes during the progression of cachexia in *Apc^Min/+^* mouse. The initiation of cachexia is associated with an increase in circulating IL-6, reduction in muscle IGF-1 expression, muscle protein synthesis and increased ubiquitin dependent proteolysis. These changes were independent of alterations in gonadal size or function. During severe cachexia, the onset of hypogonadism corresponds with a further increase in circulating IL-6 and the rise in endotoxemia. Muscle protein synthesis and IGF-1 expression are suppressed further while AMPK activation is increased. As for protein degradation, severe cachexia brings a further increase in ubiquitin-dependent proteolytic activity and the activation of autophagy-related proteolysis. Arrows indicate direction of change. Darker arrows indicate a further increase or decrease during the transition to severe cachexia.

The gradual development of tumor load and muscle wasting makes the *Apc^Min/+^* mouse an excellent model to investigate important perturbations associated with progression of cachexia (Baltgalvis et al., 2009; White et al., 2011b). We have summarized how the onset of hypogonadism relates to the progression of cachexia in the *Apc^Min/+^* mouse (Fig. 6). The development of hypogonadism is associated with the transition to severe weight loss in addition to marked reduction in muscle IGF-1 and increased proteolysis (White et al., 2011b). Furthermore, the onset of hypogonadism corresponds with increased endotoxin in the *Apc^Min/+^* mouse which could have a direct role in the development of hypogonadism (Fig. 6).

## Discussion

The development of hypogonadism in the male cachectic cancer patient has been clearly associated with increased disease severity and a poor prognosis (Del Fabbro et al., 2010). Decreased testosterone production has the potential to contribute to the overall suppression of the anabolic environment with cachexia that accelerates muscle mass loss. Currently, the contribution of hypogonadism to inflammation and insulin resistance related mechanisms that regulate muscle mass loss during cancer cachexia is not well understood. The lack of physiological models to examine cancer cachexia associated hypogonadism has created a substantial barrier for the examination of this regulation. Our current study demonstrates that the male *Apc^Min/+^* mouse is a viable model for the analysis of hypogonadal regulation of muscle mass during cancer cachexia. We demonstrate that reduced testes mass is associated with cachexia development and volitional grip strength loss in the *Apc^Min/+^* mouse. While testosterone levels are normal in weight stable and pre-cachectic *Apc^Min/+^* mice, body weight loss in cachectic mice was correlated with reduced circulating testosterone. Additionally, testosterone loss is associated with a reduction in skeletal muscle mass. Testes cellular regulation was altered with cachexia. Testes STAT3 activation and Bax protein expression were induced, while androgen receptor protein expression was suppressed with the progression of cachexia. We have previously established the importance of circulating IL-6 for cachexia development in the *Apc^Min/+^* mouse. However, IL-6 over-expression was not sufficient to reduce testes size in previously weight stable *Apc^Min/+^* mice, even though it is sufficient to induce weight loss. Our results point to a regulation of hypogonadism by IL-6, as IL-6 signaling inhibition after the initiation of weight loss attenuated the decrease in circulating testosterone and testes mass when compared to PBS treated mice.

Hypogonadism is not diagnosed in all cancers, but strongly associated with the development of cachexia (Burney et al., 2012). Furthermore, the severity of testosterone loss is associated with decreased survival rate in male cancer patients (Del Fabbro et al., 2010). While several studies have shown a reduction in circulating testosterone in patients with cancer cachexia, (Burney et al., 2012; Del Fabbro et al., 2010) data from rodent cancer models are limited. Here we report the *Apc^Min/+^* mouse has a reduction in circulating testosterone corresponding to the progression of cachexia severity. Despite polyp formation early in adulthood, (Mehl et al., 2005) testosterone levels in the *Apc^Min/+^* mouse are maintained until the later stages of cachexia, which is similar to human cachexia. The reduction in testosterone shown in the *Apc^Min/+^* mouse parallels the reduction in anabolic signaling we have observed in cachectic skeletal muscle (White et al., 2011b). In tumor bearing rats, there was no change in circulating testosterone despite the development of cachexia (Donatto et al., 2013) suggesting not all rodent models of experimental cachexia develop hypogonadism. Although strong correlations exist between testosterone loss and muscle wasting, the mechanistic role of hypogonadism during cancer cachexia warrants further investigation.

We have recently investigated the regulation of disrupted muscle protein turnover during the progression of cachexia in the *Apc^Min/+^* mouse (White et al., 2011b). We observed a reduction in protein synthesis and related mTOR signaling during initial stages of body weight loss then a progressive decrease in synthesis with further loss in body weight. In addition, we observed an increase in muscle atrogenes, atrogin1 and Murf1, during the progression of cachexia (White et al., 2011b). In the current study we report testosterone levels are not reduced during the initial stages of cancer cachexia, which suggests testosterone independent effects for the suppression of anabolic signaling at the initiation of cachexia. However, we found the progressive decrease in muscle protein synthesis and mass to be associated with altered testosterone levels. Previous experiments have shown the use of an IL-6 receptor antibody was effective in rescuing muscle wasting yet had no effect on protein synthesis (White et al., 2011b). Further investigation is needed to determine if the reduction in testosterone limits the anabolic recovery during the later stages of cachexia. Testosterone availability has direct effects on muscle anabolic signaling (White et al., 2012). Castration-induced androgen withdrawal resulted in a reduction in protein synthesis and related signaling along with an increase in gene expression related to atrogenes and the mTOR inhibitor REDD1. These findings suggest the reduction in circulating testosterone during cachexia could reduce intrinsic anabolic signaling and further suppress muscle anabolism through suppression of mTOR and consequent inhibition of protein synthesis during cachexia.

A disruption in muscle oxidative metabolism related to mitochondrial turnover is also observed during the progression of cachexia (White et al., 2011a; White et al., 2013). Recent evidence supports the regulatory interaction between mitochondrial function and protein turnover (Romanello et al., 2010). We have previously shown the *Apc^Min/+^* mouse has a reduction in muscle mitochondrial content and dysfunction in mitochondrial dynamics during the later stages of cachexia (White et al., 2011b). In addition we have reported a marked reduction in voluntary exercise in the cachectic *Apc^Min/+^* mouse (Carson and Baltgalvis, 2010; Puppa et al., 2011b); however, forced treadmill training during IL-6 induced cachexia in the *Apc^Min/+^* mouse is able to increase muscle oxidative capacity and prevent weight loss (Puppa et al., 2011b). Interestingly the same effects on muscle oxidative gene expression and physical activity can be found during testosterone withdrawal (Ibebunjo et al., 2011; White et al., 2012) suggesting hypogonadism during cachexia may also play a role in the muscle mitochondrial dysfunction and decreased exercise capacity.

There are 2 primary characterizations of hypogonadism. Primary hypogonadism involves dysfunction at the gonads and secondary hypogonadism involved dysfunction at the level of the pituitary/GNRH axis. Before the development of cachexia there are no differences in testes size, however in the cachectic *Apc^Min/+^* mouse, there is evidence of testicular atrophy which correspond to increases in testes apoptosis and STAT activation. The reduction in testes size correlates strongly with the reduction in testosterone, muscle mass and functional strength. In this study we do not have measurements of LH and FSH, making it difficult to determine the type of hypogonadism; however, there is clear gonadal dysfunction in terms of loss of mass and testosterone production. Furthermore, androgen receptor expression is decreased in the testes showing a reduction in androgen sensitivity. The loss of androgen signaling could also be a mechanism behind the induction in testes apoptosis. These data clearly show testicular dysfunction in the *Apc^Min/+^* mouse, which correspond to the reduction in testosterone and muscle wasting. Further studies are needed to determine whether hormonal imbalance and/or inflammation are responsible for testicular atrophy and cellular dysfunction.

The development of cachexia in the *Apc^Min/+^* mouse is dependent on the cytokine IL-6 (Baltgalvis et al., 2008). Circulating levels of IL-6 are highest during the later stages of cachexia in the *Apc^Min/+^* mouse (White et al., 2011b) similar to when we observe the onset of hypogonadism. IL-6 has shown to disrupt the pituitary-testicular axis in humans (Tsigos et al., 1999). In addition, circulating IL-6 correlates with the reduction in testosterone in patients with cancer cachexia (Garcia et al., 2006). In the current study, we show two weeks of IL-6 over-expression did not alter circulating testosterone or testes size. These results indicate IL-6 does not directly cause a suppression of testosterone or testes atrophy in the *Apc^Min/+^* mouse. Administration of recombinant IL-6 to human subjects showed a reduction in testosterone production during the initial days of treatment, then a return to baseline after one week (Tsigos et al., 1999). There is the possibility that the 2-weeks of IL-6 over-expression in the *Apc^Min/+^* mouse was too long to see the transient reduction in testosterone. In contrast to the acute effects of IL-6, inhibition of IL-6 signaling in *Apc^Min/+^* mice in the initial stages of weight loss partially rescued the loss of testosterone and testes atrophy during the progression of cachexia. Although we can not definitely conclude whether IL-6 acts directly or indirectly in the development of hypogonadism, it appear IL-6 is necessary but not sufficient to induced the hypogonadal condition in the *Apc^Min/+^*mouse.

Another possible factor contributing to the onset of hypogonadism during late stage cachexia is endotoxemia. We have previously shown endotoxemia in cachectic *Apc^Min/+^*mice which is associated with gut barrier dysfunction (Puppa et al., 2011a). Although endotoxemia is observed during severe cachexia when IL-6 levels peak, endotoxin can independently induce hypogonadism. Sam et al. reported septic rats had a reduction in circulating testosterone and testicular dysfunction (Sam et al., 1999). Furthermore, treatment of endotoxin and interferon-γ to C2C12 myotubes has been shown to mimic several signaling events we observed in muscle during severe cachexia including the reduction in phosphorylated mTOR and p70S6k and the increase in phosphorylated raptor and activation of AMPK (Frost et al., 2009). The role of endotoxemia in the onset of hypogonadism and other cachexia-related symptoms is unclear and requires further investigation.

In summary, we are the first to report the *Apc^Min/+^*mouse is a useful model of hypogonadism during cancer cachexia. The *Apc^Min/+^*mouse shows a reduction in testosterone and testicular atrophy as the severity of cachexia increases. In addition to testes atrophy, we report an increase in apoptosis and STAT activation in testes during the development of hypogonadism. Acute over-expression of IL-6 did not decrease testosterone or testes size in the *Apc^Min/+^*mouse. However, inhibition of IL-6 signaling through an IL-6 receptor antibody attenuated the decrease in testosterone and testicular atrophy in mice initiating weight loss. Establishing the *Apc^Min/+^*mouse as a model for hypogonadal cancer cachexia will allow further investigation on the role of testosterone on muscle wasting.

## Materials and Methods

### Animals

The University of South Carolina's Institutional Animal Care and Use Committee approved all animal experimentation in this study. ApcMin/+ mice on a C57BL/6 background were originally purchased from Jackson Laboratories (Bar Harbor, ME) and bred at the University of South Carolina's Animal Resource Facility. Male ApcMin/+ (*n* = 21) mice between 14 and 20 weeks of age were group housed and sacrificed at ages that provided stratification of body weight loss to allow the study of the progression of cachexia. The 4 groups used in this study were weight stable (WS; *n* = 5), initial (≤5%; *n* = 6), moderate (6–19%; *n* = 4), and severe (≥20%; *n* = 6) degrees of body weight loss, when compared to peak body weight. To block the progression of cachexia, a separate set of ApcMin/+ mice were treated with an IL-6 receptor antibody (*n* = 5) or PBS control (*n* = 7) for two weeks, starting after the onset of cachexia (16 weeks). Wild-type C57BL/6 controls were also treated with the IL-6 receptor antibody (*n* = 6) or Phosphate Buffered Saline (PBS) control (*n* = 6) at 16 weeks. A subset of ApcMin/+ mice were electroporated to transfect a control vector (Vector, *n* = 5) or IL-6 over-expressing vector (*n* = 6) at 12 weeks of age, see methods below. The room was maintained on a 12:12 light: dark cycle with the light period starting at 0700. Mice were provided standard rodent chow (Harlan Teklad Rodent Diet, #8604, Madison, WI) and water ad libitum.

### IL-6 receptor antibody administration

IL-6 receptor antibody was administered as previously described (White et al., 2013). The MR16-1 IL-6 receptor antibody was a generous gift from Chugai Pharmaceutical Co. Ltd, Tokyo, Japan. The antibody was administered at a dose of 300 µg/mouse in PBS by intraperitoneal injection once every three days for two weeks starting at 16 weeks of age. PBS was injected as a control vehicle.

### IL-6 over-expression

*In vivo* intramuscular electroporation of an IL-6 plasmid was used to increase circulating IL-6 levels in mice as previously described (White et al., 2013). The quadriceps muscle was used as a vessel to produce IL-6 and secrete it into circulation, and was not used for any analyses in the study. The gastrocnemius muscle used in the study was not subjected to electroporation. Briefly, mice were anesthetized with a 2% mixture of isoflurane and oxygen (1 L/min). The leg was shaved, and a small incision was made over the quadriceps muscle. Fat was dissected away from the muscle, and mice were injected with 50 µg of the IL-6 plasmid driven by the CMV promoter, or empty control vector, into the quadriceps muscle in a 50 µl volume of phosphate-buffered saline (PBS). A series of eight 50 ms, 100 V pulses was used to promote uptake of the plasmid into myofibers, and then the incision was closed with a wound clip. Both vector control and + IL-6 groups received the appropriate plasmid starting at 12 weeks of age. Mice were sacrificed after 2 weeks after IL-6 over-expression.

### Tissue collection

Mice were given a subcutaneous injection of ketamine/xylazine/acepromazine cocktail (1.4 ml/kg BW) before the gastrocnemius and testes were dissected. Tissues were rinsed in PBS, weighed, snap frozen in liquid nitrogen and stored at −80°C until further analysis.

### Serum testosterone

Testosterone was measured by the commercial testosterone EIA kit (Cayman Chemical, Ann Arbor, MI) as per the manufacturer's instructions.

### Grip testing

Forelimb grip strength was assessed at the end of the study. Each mouse was allowed to grab a bar attached to a force transducer as it was pulled by the tail horizontally away from the bar (Model 1027CSM; Columbus Instrument Co., Columbus, Ohio) (Smith et al., 1995). Five repetitions with a 5-s pause between each were averaged to determine grip strength for each mouse.

### Myofibrillar protein synthesis

Myofibrillar protein synthesis measurements were performed as previously described (Welle et al., 2006; White et al., 2011c). Gastrocnemius muscle samples were homogenized in 1 ml water. Myofibrils and other insoluble proteins were pelleted by centrifugation, and the supernatants containing free amino acids were used to determine the ratio of free 2H5-phenylalanine (m/z 239 fragment) to endogenous (unlabeled) phenylalanine (m/z 234 fragment). The ratios were determined by GC-mass spectrometric analysis of the t-butyldimethylsilyl derivatives of these amino acids. Myofibrillar proteins were washed, hydrolyzed, and analyzed for 2H5-phenylalanine enrichment by monitoring the m/z 237 and 239 fragments.

The fractional rate of myofibrillar synthesis, % per day, was calculated as the % enrichment of tracer in the hydrolysate of myofibrillar protein, divided by the tracer enrichment in the free amino acid pool of muscle tissue. Myofibrillar protein enrichment was determined from the m/z 237 and m/z 239 ions because the lightest isotopomer (m/z 234) saturated the MS detector. The myofibrillar/free enrichment ratio was multiplied by 48 to obtain %/day values because tracer incorporation occurred over a period of 30 min.

### Western blotting

Western blot analysis was performed as previously described (White et al., 2009). Briefly, frozen gastrocnemius muscle was homogenized in Mueller buffer and protein concentration determined by the Bradford method (Bradford, 1976). Crude muscle homogenate 40 µg was fractionated on 6–15% SDS-polyacrylamide gels. Gels were transferred to PVDF membranes overnight. Membranes were stained with Ponceau to verify equal loading of each gel. Membranes were blocked overnight in 5% non-fat milk in Tris-buffered saline with 0.1% Tween-20 (TBS-T). Primary antibodies for p-STAT3 (Tyr705), STAT3, (Cell signaling), Bax and androgen receptor (Santa Cruz) were diluted 1:1000 to 1:500 in 5% milk in TBS-T followed by 1 hour incubation with membranes at room temperature. Anti-rabbit IgG horseradish-peroxidase conjugated secondary antibody (Cell Signaling) was incubated with the membranes at 1:2000 dilutions for 1 hour in 5% milk in TBS-T. Enhanced chemiluminescence (ECL) (GE Healthcare Life Sciences, Piscataway, NJ) was used to visualize the antibody-antigen interactions. Images were digitally scanned and blots were quantified by densitometry using scientific imaging software (Scion Image, Frederick, MD).

### Statistical analysis

A one way ANOVA was used to determine differences in ApcMin/+ mice with varying degrees of body weight loss. A two way ANOVA was used to determine differences between genotype and IL-6 receptor antibody treatment. Post-hoc analyses were performed with Student-Newman-Keuls methods. The Pearson correlation coefficient was used to determine linearity throughout the manuscript. T-tests were used to compare control vector and IL-6 over-expression groups. A pre-planned t-test was used to compare testes weight between PBS and IL-6 receptor antibody treated mice. Significance was set at *P*<0.05.
